# Biological Stoichiometry in Tumor Micro-environments

**DOI:** 10.1371/journal.pone.0051844

**Published:** 2013-01-22

**Authors:** Irina Kareva

**Affiliations:** Center of Cancer Systems Biology, Steward St. Elizabeth's Medical Center, Tufts University School of Medicine, Boston, Massachusetts, United States of America; Glasgow University, United Kingdom

## Abstract

Tumors can be viewed as evolving ecological systems, in which heterogeneous populations of cancer cells compete with each other and somatic cells for space and nutrients within the ecosystem of the human body. According to the growth rate hypothesis (GRH), increased phosphorus availability in an ecosystem, such as the tumor micro-environment, may promote selection within the tumor for a more proliferative and thus potentially more malignant phenotype. The applicability of the GRH to tumor growth is evaluated using a mathematical model, which suggests that limiting phosphorus availability might promote intercellular competition within a tumor, and thereby delay disease progression. It is also shown that a tumor can respond differently to changes in its micro-environment depending on the initial distribution of clones within the tumor, regardless of its initial size. This suggests that composition of the tumor as a whole needs to be evaluated in order to maximize the efficacy of therapy.

## Introduction

Cancer can be viewed as an ecological system: a tumor is a heterogeneous population of cells, including both malignant and non-transformed somatic cells of the stroma, which compete for space and nutrients within the dynamic environment of the human body [1]–[4]. Whatever intrinsic properties each individual cell may have acquired through mutations – e.g. propensity for increased proliferation, low mortality or both – cancer cells must first allocate nutrients to survival and physiological cell maintenance before they can proliferate, invade surrounding tissues and metastasize.

High-energy electrons of organic carbon are the main energy source for all cells. Moreover, both carbon (C) and phosphorus (P) are among the primary components of cell building materials. Specifically, glycolytic intermediates obtained from the breakdown of glucose are used for the biosynthesis of nucleic acids via the pentose phosphate pathway. This process involves generating NADPH, which is then used for fatty acid synthesis, and ribose-5-phosphate, which is used for synthesis of nucleotides and nucleic acids [5]. These considerations underlie the growth rate hypothesis (GRH), which suggests that highly proliferative cells are characterized by relatively low C∶P stoichiometry due to their up-regulation of the P-rich ribosomes needed to support reproduction [6]–[8]. Applied to cancer, the GRH predicts that tumors consisting of highly proliferative clones should be characterized by low C∶P ratios, and their growth rates should increase with increased access to P. The second prediction has been experimentally verified [9], [10]; the first prediction appears to be supported in cancers of the colon and the lung but not in the kidney or liver, suggesting the possibility that the micro-environment in these two organs may favor selection of clones with propensity for apoptosis evasion rather than increased proliferative potential [11].

As adaptive systems composed of interdependent, genetically diverse cells, tumors, like other ecosystems, are likely to be too complex to be controlled directly. However, manipulation of the tumor environment, both locally (micro-environment) and globally (whole-body), may allow for directing tumor evolution toward a more desirable clinical outcome. Cancer cells can maximize their fitness, that is, overall growth rates measured as the difference between birth and death rates, by allocating available resources (such as carbon and phosphorus) either towards rapid proliferation (r-clones) or first allocating nutrients towards increased survival and evasion of apoptosis, thus proliferating slowly (s-clones). Within this construct, tumors can be viewed as genetically and phenotypically heterogeneous populations of cells in which clonal lineages vary by their genetic ‘choice’ of strategy along this selective axis (rapid proliferation vs slow proliferation but increased survival). Depending on the selective pressures that the population has experienced in the past, at any given time the tumor can be monomorphic (all cells use the same strategy) or polymorphic (multiple clones using different strategies). Selective pressures favoring rapid or slow proliferation vary with micro-environment (local selection pressure) and the host's general physiological condition (global selection pressure). The purpose of this work is to provide a modeling framework that will enable further investigation of the GRH as applied to cancer, extending the work done by Elser and colleagues [7], [11]. Specifically, the GRH is used as a theoretical foundation to construct a mathematical model of tumor evolution from an ecological perspective, which allows evaluating the effect of C∶P stoichiometry on natural selection within a tumor in response to the micro-environmental conditions. The model is then used to assess whether manipulation of this ratio can be exploited in to direct evolution of a tumor away from a rapidly proliferating cell phenotype.

## Materials and Methods

### Model Description

**Figure 1:**
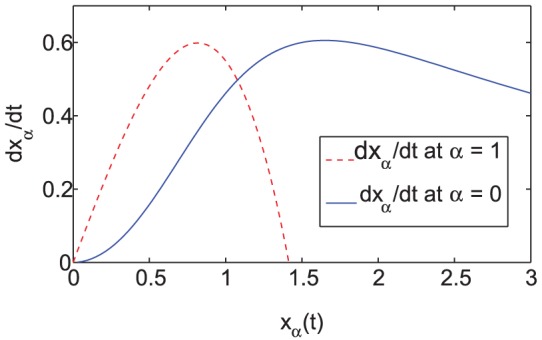
Schematic representation of the interaction between the two growth strategies. Depending on the initial composition of the population (the number of clones conforming to one strategy over the other), either s-clones or r-clones have higher fitness (larger value of 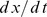). At the intersection of the two curves, neither strategy gives the clones an advantage, regardless of the distribution of clones in the population (i.e., regardless of the value of 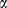).

Suppose cancer cells require some resource or combination of resources (to be made explicit below) for both proliferation and maintenance physiology. The amount of resource in the environment at time 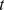 is denoted as 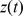. In this model, the evolutionary strategy of how cell clones partition their resources, either for rapid proliferation or slow proliferation but increased survival, is henceforth referred to as r-strategy and s-strategy, respectively. Per capita reproduction among r-clones is assumed to be 
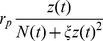
, where 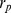 is their (constant) intrinsic division rate, and 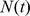 is the total number of cells in the tumor; since r-clones allocate only a minimum of resources to maintenance, it is assumed that they die at some constant per capita rate 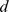. The functional form for the growth term is chosen in such a way as to incorporate the assumptions that 1) all the available resource is directly invested into increasing the number of cells, and 2) proliferation is inhibited by excess resource, as has been observed in other ecosystems (for phosphorus, for example) [12], which is accounted for in the model with 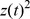. In contrast, the per capita growth rate of s-clones takes the logistic form, 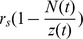, where 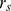 is the intrinsic rate of increase for s-clones and the carrying capacity is proportional to the amount of resource. This functional form allows for the incorporation of the proposed fundamental difference in resource partitioning strategies between r- and s-clones, while preserving the overall shape of the growth curve (see Figure 1). Noticeably, the functional form of the growth functions for the two clone types was chosen specifically to parallel the functions used in corresponding ecological literature, and specifically, in [13].

Now let us introduce parameter 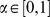, such that a given clone allocates a constant proportion, 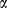, of its resources to maintenance physiology at the expense of rapid proliferation (s-strategy), while the remaining 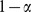 is allocated to proliferation (r-strategy). Therefore, the difference in growth rates among r-clones is due entirely to variation in amount of resource they have been able to acquire.

Now suppose that each cell can adopt a mixed strategy in its resource allocation, allotting different proportions of its resources towards rapid proliferation and physiological maintenance; such a cell would then be characterized by its own intrinsic value of parameter 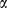 and is henceforth denoted as 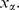 A collection of cells, characterized by the same value of 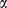 is referred to here as 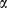-clone. Let 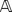 denote the range of possible values of parameter 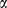. The total population size can then be calculated as 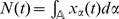. The dynamics of any single clone engaging in a mixed strategy is thus described by
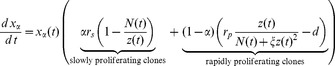
(1)and the dynamics of the entire population is given by 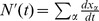. Selection pressures on the various strategies vary with environmental conditions, i.e., with changes in 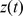. The consequence of this selection is reflected in the per capita growth rate for that clone, *viz.* 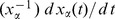. That is, a clone with many cells may have a higher overall growth rate but may still be declining relative to another clone with a higher per capita growth rate but with fewer cells. The transitional regime, in which neither strategy (rapid proliferation or slow proliferation but increased survival) holds a competitive advantage, occurs when 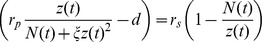.

Schematically, this interaction is depicted in Figure 1, where the growth functions are plotted for both ‘pure’ strategies relative to each other. As can be seen, depending on the composition of the population (the number of clones conforming to one strategy over the other) at each time point, either ‘pure’ s-clones or ‘pure’ r-clones have higher fitness (larger value of 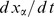 per 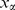). At the intersection of the two curves, neither strategy provides either clone type with a competitive advantage in terms of higher growth rate, regardless of the distribution of clones in the population (i.e., regardless of the value of 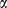). In order to appropriately address the question of how the population composition will change due to natural selection in response to micro-environmental perturbations, and specifically, in response to changes in phosphorus availability, a possibility of ‘intermediate’ strategies needs to be introduced, which is done in the following sections.

### Composite resource and biological stoichiometry

The amount of a given elemental resource available to an organism is best measured not by its absolute abundance but by its quantity relative to other elements making up the organism's biomass, since elemental ratios in biomass must remain balanced (reviewed extensively in [6]; see also [8], [12], [14]). Here 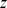 is recast as the amount of exploitable C relative to P. Carbon is usable only if there is sufficient P to build a cellular apparatus (primarily ribosomes) to process it. Therefore, we assume that 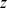 is a saturating function of the C∶P ratio, namely 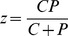.

### The full model

In our model, P traffics between intra- and extracellular compartments within the tumor. Arterial P delivery occurs at rate 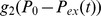 , where 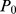 is the P concentration in arterial blood, and 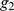 is the capillary permeability constant. The term is chosen based on standard chemostat models (see, for instance, [15]). Cells absorb this interstitial P via a saturating, bidirectional membrane transporter (essentially facilitated diffusion) at a rate 
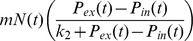
, where 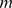 represents maximal per cell transport rate, and 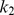 is the half-saturation constant for the transporter. Intracellular P reenters the interstitium at rate 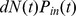 when cells die. We assume that specialist cells within a clone vary in resource uptake rate; therefore, 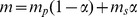, where 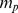 and 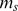 are the maximal nutrient uptake rates for r-clones and s-clones specialists, respectively. The absolute amounts of nutrient are then recalculated into concentrations, yielding the following system of equations (see Appendix S1 for complete derivation):
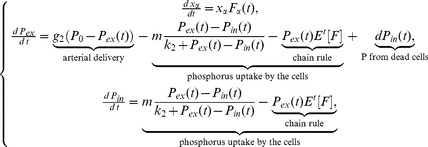
(2)where 
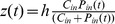
,
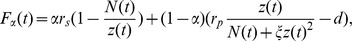


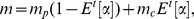


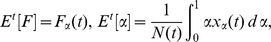
assuming 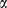 is continuous.

Since the goal of the proposed model is to evaluate how fluctuations in external nutrient supply, and particularly variations of parameter 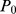, affect the distribution of cell phenotypes within a tumor, a way to measure how the phenotypic distribution changes over time is needed. Assume that different clones within the tumor are represented in different proportions depending on the value of parameter 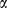, falling within some initial known distribution. If the choice of strategy with respect to interactions with the resource (using available resources for rapid proliferation or for slow proliferation but increased survival) affects fitness, then each subpopulation of clones, characterized by 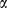, will grow at different rates. Consequently, the distribution of clones within the entire cell population will be changing over time, and so will the expected value of 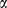. In its current form, System (2) is infinitely dimensional. However, let us introduce auxiliary ‘keystone’ variables 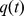 and 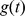 such that
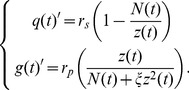
(3)


Then the equation for the total population of cells 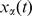 can be rewritten as
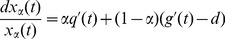
(4)Integrating Equation (4) yields the following explicit expression for 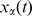 in terms of keystone variables 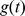 and 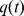:
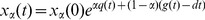
(5)Then the full population size becomes
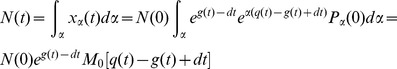
(6)where 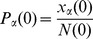 and 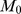 is the moment generating function (mgf) of 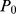, so that the final distribution of clones over time becomes
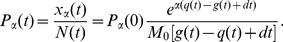
(7)With these transformations, the otherwise infinitely-dimensional System (2) can now be rewritten as a system of four non-autonomous ODEs:
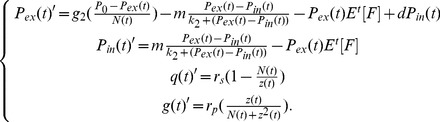
(8)The mean value of parameter 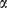 is given by
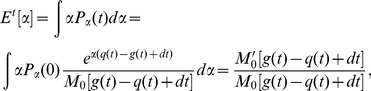
(9)where 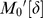 is the derivative of the moment generating function at 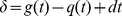; the total population size is defined above. Population composition with respect to strategy choice can now be tracked through changes in the mean of 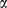 such that the higher the value of 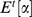, the more s-clones there are in the population, and therefore fewer cells remain that will invest primarily into rapid proliferation.

Note that System (8) is non-autonomous, rendering standard bifurcation analysis techniques inapplicable. However, sensitivity analysis can be performed in order to evaluate, variations in which parameters would cause the largest changes in the overall system dynamics, thus guiding further investigations.

#### Parameter estimation

The proposed model is characterized by three variables and thirteen parameters. 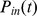 and 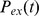 are the concentrations of intracellular and extracellular phosphorus in the tumor micro-environment (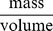). 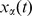 is the volume, occupied by a set of cells, characterized by intrinsic value of 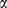 (we choose to case 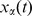 in the units of volume and not mass of cells in order to be able to model nutrient uptake as a difference of concentrations of mass/volume), and 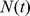 is the total volume occupied by all the cell clones.

The key parameter 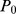, which accounts for extrinsic phosphorus inflow, was estimated based on the following considerations, outlined also in [7]: if we assume a dietary intake of approximately 210 mg of phosphorus per day for a healthy 70 kg person, then the daily share of P for a 10 kg organ is approximately 30 mg. The concentration of intracellular carbon 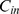 is held constant; its value is estimated based on the normal homeostatically maintained concentration of glucose in the blood, which is 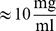. The per capita growth and death rates of cell clones, as well as saturation constant 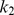 and the phosphorus ‘gating’ constant 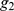, were taken from a previously published model [7]; the values for the growth and nutrient uptake rates of s-clones (parameters 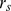 and 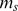) were taken as relative to the corresponding values of r-clones (parameters 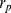 and 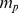), based on theoretical considerations outlined in the previous sections. Parameters pertaining to population heterogeneity, and specifically to the initial distributions, such as parameters 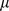 and 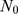, were chosen arbitrarily due to a lack of data on the possible distributions of clones with respect to resource uptake ‘strategy’ within tumors; however, with a possible development of appropriate measurement techniques, such data could theoretically be obtained. The values for scaling constants 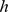 and 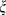 were also chosen arbitrarily due to lack of data.

All variables and parameters with corresponding units are summarized in Table 1.

**Table 1:** Summary, brief description and range of all parameters in System (2).

	Meaning	Range	Units
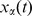	Volume, occupied by a set of cells, characterized by intrinsic value of 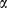	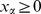	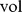
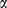	Proportion of slowly proliferating clones (s-clones) in the population	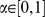	n/a
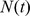	Volume, occupied by the total cell population size: 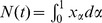	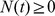	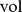
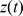	Amount of composite resource: 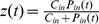	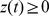	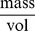
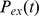	Concentration of extracellular phosphorus	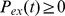	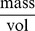
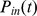	Concentration of intracellular phosphorus	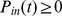	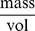
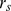	Intrinsic growth rate of slowly proliferating cells (s-clones)	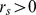	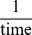
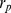	Intrinsic growth rate of rapidly proliferating cells (r-clones)	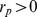	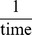
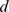	Per capita cell death rate	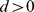	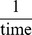
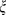	Resource uptake conversion factor for r-clones	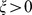	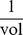
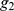	Scaling constant for P inflow	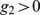	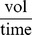
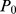	Gradient constant for amount of arterial P inflow	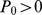	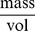
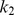	Half saturation constant for P uptake by the cells	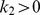	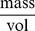
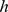	Scaling constant for optimal C∶P ratio	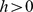	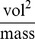
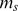	Phosphorus uptake rate of s-clones	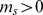	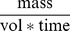
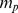	Phosphorus uptake rate of r-clones	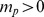	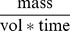
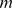	Total carbon uptake rate: 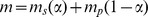	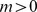	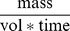
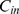	Concentration of intracellular carbon (constant)	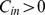	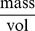

#### Sensitivity analysis

Parameter values, estimated above, describe average tumor growth. Within each parameter, however, there can be significant variability, which is reflected in patient-specific clinical disease courses of tumors of the same organ. Moreover, low dimensionality of the proposed model may result in a compression of several parameter values into the same parameter, introducing further variability. In order to evaluate, which parameters in System (8) would have the largest impact on the overall system dynamics, sensitivity analysis was performed. To measure global sensitivity, it was assumed that each parameter is perturbed by a uniformly distributed random variable within the range of 25% of the initial parameter value. The sensitivity indices, which are defined as fractions of total output variance generated by the uncertainty in the respective parameter value, were calculated using the Fourier Amplitude Sensitivity Test (FAST) method [16], an approach that allows investigating the effect of large, concurrent perturbations in model parameters.

**Figure 2:**
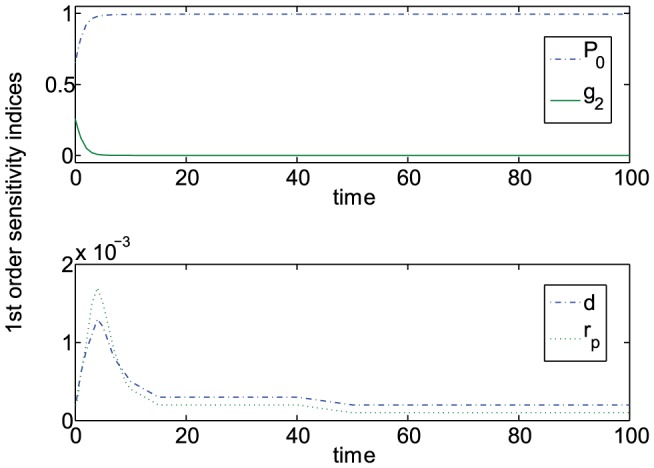
First order sensitivity indices defined as the fraction of the total variance in system behavior caused by the variation in each parameter value. Calculations were performed for a 13-parameter vector V = [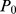, 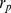, 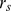, 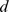, 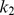, 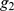, 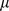, 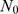, 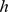, 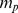, 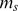, 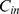, 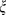], using the FAST method and under the assumption that parameters are varied uniformly by no more than 25%. Due to vast differences in relative importance of different parameters, the results are reported on two separate graphs; only parameters with sensitivity indices over 1% are reported.

First, the sensitivity of concurrent perturbations to all the thirteen parameters in System (8), namely, 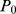, 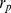, 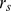, 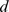, 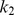, 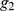, 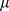, 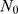, 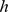, 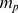, 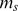, 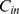 and 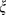. was investigated. The results, albeit expected, proved to be stronger than anticipated, revealing that by time 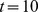, over 90% percent of variation in the final outcome of system behavior is due to uncertainty in the rate of phosphorus inflow, given by parameter 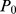 (see Figure 2a). The only other parameter that also caused significant variations in overall system behavior at the initial stages of tumor growth was parameter 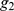, a ‘gating’ parameter, which accounts for diffusion of extracellular phosphorus into and out of the tumor micro-environment. Relative contributions of some of the other parameters are shown in Figure 2b.

**Figure 3:**
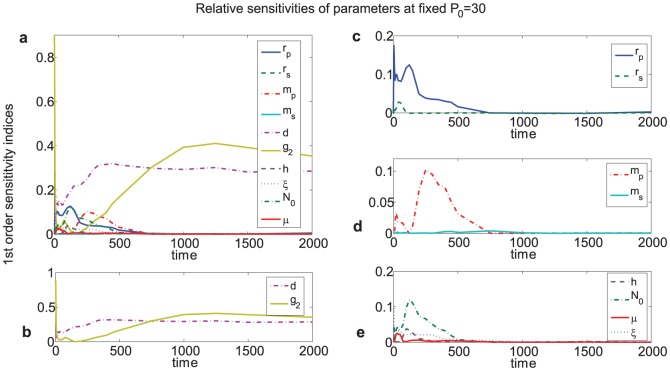
First order sensitivity indices defined as the fraction of the total variance in system behavior caused by the variation in each parameter value under constant 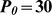. Calculations were performed for a 12-parameter vector V = [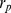, 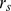, 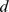, 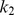, 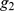, 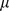, 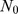, 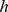, 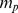, 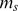, 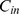, 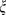], using the FAST method and under the assumption that parameters are varied uniformly by no more than 25%. Due to vast differences in relative importance of different parameters, the results are reported (a) together and (b–e) on four separate graphs, grouped by the order of magnitude of the corresponding sensitivity index; only parameters with sensitivity indices over 1% are reported.

Next, we wanted to evaluate the relative influence of other parameters under the condition of constant P inflow. For this purpose, 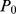 was fixed at a value of 30, the estimated ‘normal’ inflow of extracellular phosphorus into the tumor micro-environment, effectively reducing the parameter vector to twelve parameters. As can be seen in Figure 3a, the parameters that consistently have the largest influence on overall system behavior are parameter 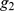, the phosphorus ‘gating’ parameter and parameter 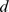, the natural death rate of highly proliferative tumor cells (Figure 3b). Moreover, the relative influence of different parameters seems to be quite variable over time. As shown in Figure 3c, relative per capita growth rates of both clone types, namely, parameters 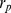 and 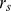, are of vital importance in the initial stages of tumor growth, before 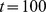, while nutrient uptake rates 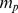 and 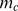 become important later on, supposedly when the number of cells is large enough (Figure 3 d) for the dynamics to become primarily influenced by active competition for nutrients, and specifically to this case, phosphorus. Finally, it is also important to note that parameters associated with modeling parametric heterogeneity in the population, namely 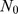 and 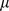, which determine the initial composition of the population and the total population size, are also of vital importance in the initial stages of tumor growth.

In addition to parameter 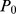, parameters that pertain to interactions between two clone types, i.e., 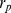 and 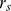, 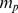 and 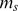, and 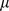, and their effect on tumor dynamics and composition will be the focus of the following section.

## Results

First, the hypothesis that increased phosphorus inflow could shift population composition toward a more rapidly proliferating phenotype due to increased availability of building materials for DNA, RNA and ribosomes [11], [12] was evaluated. Changes in population composition were measured through the changes in the expected value of parameter 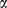 as 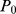 is increased. The higher the value of 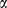, the larger the proportion of s-clones is present in the population. The initial conditions were taken to be 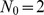, 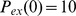, 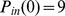, 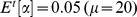, 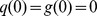 and all the parameter values are taken to be 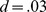, 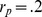, 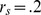, 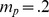 ,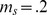, 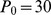, 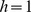, 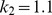, 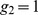, unless indicated otherwise. Numerical solutions were calculated until 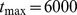. Parameter values were chosen to illustrate conceptual differences between dynamical behaviors and do not conform to any particular data set. The initial distribution was taken to be truncated exponential on the interval 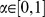. Matlab2010a code is available upon request.

**Figure 4:**
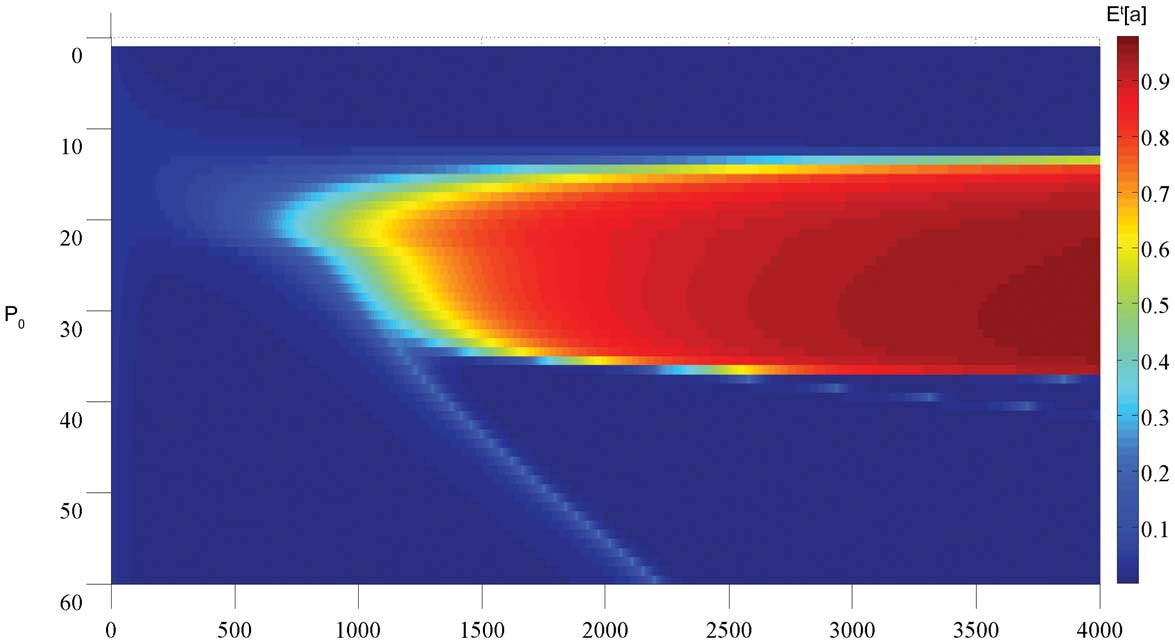
A heat map of the change in the expected value of 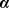 over time for different fixed 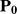; 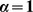 corresponds to predominance of s-clones, 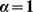 – to predominance of r-clones.

Numerical solutions to System (8) support the hypothesis that increasing availability of extracellular phosphorus indeed creates an environment that favors expansion of r-clones, which is reflected in the changes in the expected values of 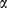. In Figure 4, one can see which clone type comes to predominate over time depending on the initial value of parameter 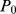. The results suggest that there in fact exists a ‘window’ of values of 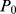 (relative to 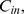 which in this model is kept constant), where slowly proliferating clones are favored over rapidly proliferating ones. Unexpectedly, when the value of 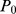 is increased beyond 30, one can additionally observe transitional regimes, as 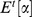 increases briefly, temporarily favoring s-clones in a population, composed primarily of r-clones. This effect could be interpreted as saltatory tumor growth [17], and can be observed in the proposed model only under the assumption of the possibility of ‘futile metabolism’ (also known as ‘the paradox of enrichment’), which predicts decline in growth rates under the conditions of nutrient over-saturation (a more detailed discussion of this phenomenon is given below) and which is captured here using a variant of the Holling type III function to describe growth rates of r-clones; noticeably, assumption of Holling type II functional form, i.e., when 
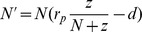
, did not predict saltatory tumor growth regardless of the concentration of extracellular phosphorus (calculations not shown).

### Differences in growth rates and nutrient uptake rates

**Figure 5:**
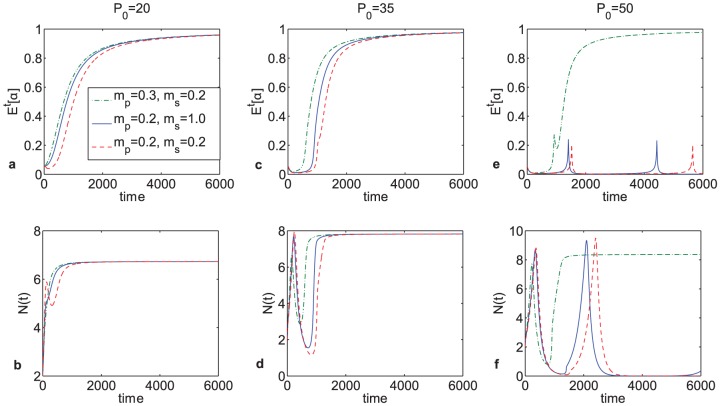
The effects of differences in nutrient uptake rates on cell population size composition at 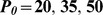. Noticeably, r-clones are either practically unaffected or at a loss regardless of the relative values of parameters 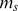 and 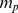, which suggests that targeting phosphorus transporters might in fact give advantage to r-clones regardless of which clone type may have this adaptation.

Next, changes in population dynamics in response to differences in nutrient uptake rates were evaluated for 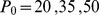. When s-clones have higher phosphorus uptake rates, population composition unexpectedly does not shift toward the predominantly slowly growing cell phenotype over time (Figure 5); this effect can be accounted for by growth limitations that are imposed by carbon availability, which in our model is kept constant. However, interestingly and counter-intuitively, higher phosphorus uptake rates do not promote selection for r-clones over time either, as can be seen on Figure 5 through the changes in 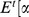] over time when 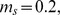 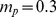. This effect can be explained in the following way: even though more r-clones take up more phosphorus, unless they have enough carbon to meet the energy demands for rapid proliferation, they cannot use it, thus engaging in a form of ‘futile metabolism’. These results suggest that under the conditions of constant glucose supply, increased 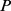 uptake rates would not give r-clones an advantage regardless of which clone type has this adaptation, and so targeting phosphorus transporters would in fact promote rapidly proliferating clones rather than inhibit their growth. Noticeably, for 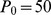, which simulates conditions that are expected to be most conducive to expansion of r-clones under the GRH, oscillations in tumor size are predicted by the model, which are typically not recorded in patients. This is most likely due to the fact that conditions of such high concentrations of 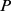 relative to 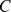 are unlikely to be observed, except after high doses of cytotoxic therapies, which would cause extensive cell death and consequent release of large amounts of intracellular phosphorus in the bloodstream (hyperphosphoremia). The model predicts that such conditions would be conducive to expansion of the rapidly proliferating cell types, and consequently this is when one could expect to see oscillations in tumor size over time, driven by changes in tumor composition.

**Figure 6:**
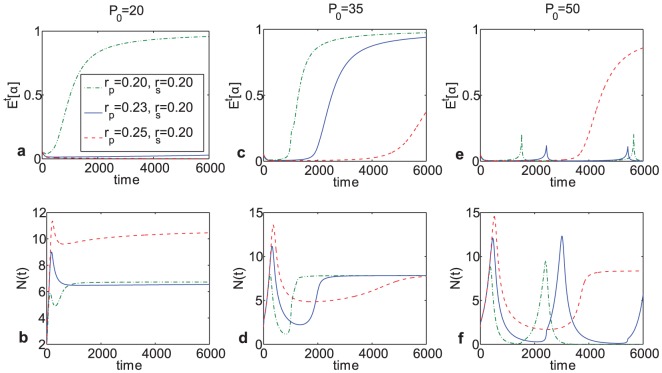
Effects of variation in intrinsic growth rates on population size and composition at 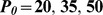. Population size does not necessarily reflect population composition, suggesting that devising treatments based solely on tumor size may not necessarily be adequate.

Next, the effect of changes in the intrinsic growth rates of either clone type on the overall population composition were evaluated for 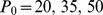. As can be seen on Figure 6, depending on the initial value of 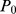, the ratio of 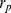 to 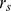 needed to be increasingly larger in order to give r-clones a sufficient competitive advantage to enable them to predominate within the population for longer periods of time. Interestingly, the overall final population size could remain the same regardless of composition, as can be clearly seen on Figure 6b for 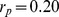 and 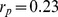. This suggests that tumor size is not necessarily a good predictor of population composition, and the distribution of clones within the tumor needs to be evaluated separately, since a tumor may respond differently to micro-environmental changes – whether it be nutrient availability or the presence of therapeutic agents – depending on the initial distribution of clones within it.

### Different initial distributions

**Figure 7:**
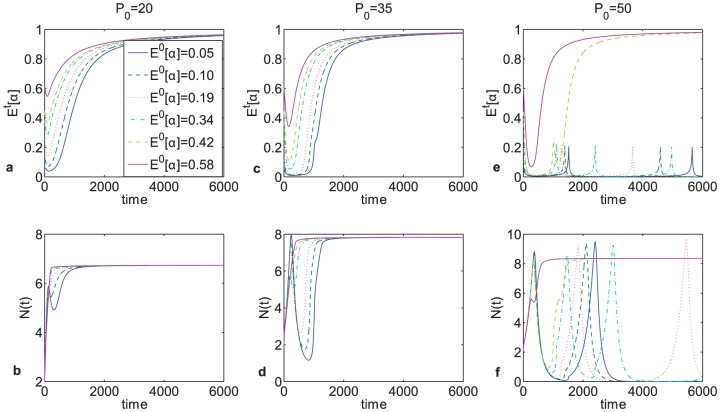
Changes in the mean of 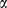 and the full population size at 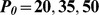. with respect to differences in initial composition of the population, reflected through varying 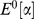. The results suggest that just knowing the state of the tumor micro-environment is not necessarily enough to be able to predict in which direction the population will evolve; the composition of tumor must also be known.

Since tumors evolve over time, the overall composition of the cell population may vary at different time points, and hence the population as a whole may respond differently to the same set of micro-environmental perturbations. This predicted effect was evaluated in the model through varying the initial distribution of clones within the population. In Figure 7, it was observed that under exactly the same set of initial conditions, the population as a whole evolved in different directions, depending on the initial distribution of cell clones in the population with respect to 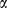. Specifically, it was shown that the higher the initial proportion of s-clones in the population, the more phosphorus was required to shift the population composition towards being dominated by a more rapidly proliferating phenotype. This is due to the fact that the direction in which the system evolves is dependent not only upon external factors but also on population composition, since cells within the population impose as much of a selective pressure on each other as is imposed on them by their extrinsic environment. Therefore, from a therapeutic point of view, in order to evaluate the extent of micro-environmental manipulation required to shift the population composition away from a more malignant phenotype, one would first need to evaluate tumor composition with respect to rapidly-proliferating vs. slowly-proliferating phenotype, i.e., using this particular characteristic as a metric by which to quantify the level of heterogeneity; naturally, other metrics would need to be used for other types of treatments, where tumor heterogeneity plays a role.

## Discussion

Tumors can be viewed as evolving adaptive ecological systems, in which heterogeneous populations of cells compete with each other and with somatic cells for limited space and nutrients. Regardless of the properties that the cells may have acquired through mutations, they first need nutrients to survive, and may adopt different strategies to achieve this goal. Depending on the specific conditions in their micro-environment, cells try to maximize their fitness by investing the available resources primarily in proliferation or in physiological maintenance, achieved at the expense of rapid proliferation. Consequently, appropriate manipulation of the tumor environment, both locally (micro-environment) and globally (whole-body), may allow to direct tumor evolution toward a more desirable clinical outcome.

Proposed here is a focus on phosphorus as one of the key elements of the cell micro-environment, and specifically, on stoichiometric ratios between phosphorus and carbon, which, according to the growth rate hypothesis (GRH), may influence the direction of system evolution over time [8]. It is hypothesized that increased availability of phosphorus in the tumor micro-environment might promote selection for more rapidly proliferating cell types and thus for a potentially more malignant tumor. The hypothesis is evaluated using a system of ordinary differential equations, which models changes in tumor composition with respect to the strategy that the cells use for resource utilization subject to changes in resource availability.

It is assumed that each cell can adopt a mixed strategy for its resource partitioning, allocating different proportions of its resources to rapid proliferation and to physiological maintenance; the extent to which each cell favors one strategy over the other is accounted for by its own intrinsic value of parameter 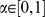, where 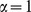 corresponds to investing the resource primarily for physiological maintenance at the expense of rapid proliferation (s-clones), and 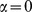 corresponds to using the resources to invest primarily in rapid proliferation (r-clones). It is assumed that different clones within the tumor are represented in different proportions depending on the value of parameter 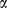, falling within some initial known distribution. Fluctuations in population composition are tracked through the changes in the mean value of 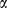 as the system evolves over time. Within the context of the proposed model, a set of cells characterized by the same value of 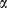 is defined as 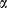-clone; fitness of each clone is defined as 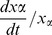.

The results of numerical computations (see Figure 4) suggest that indeed, increased inflow of extracellular phosphorus promotes a shift towards the more rapidly proliferating cell type largely in accordance to GRH. This was also experimentally suggested by [11], where it was shown that the intracellular concentration of phosphorus was indeed significantly higher in some types of tumors than in somatic tissues. The authors suggested that some tumors may be composed of cell types that use available resources primarily for rapid proliferation, as opposed to physiological maintenance, depending on the micro-environmental conditions in each particular organ of tumor origin. The evolutionary and ecological perspective on cancer development also suggests that sampling a tumor at just one time point might not give accurate information about its stage of development along the evolutionary track due to genotypic and phenotypic changes in tumor composition over time.

An interesting effect that occurred at very high concentrations of extracellular phosphorus could be observed in numerical simulations: both tumor size and composition fluctuated, but not in a steady oscillatory manner (see Figure 4, and panels (e–f) in Figures 5, 6 and 7). Instead, the population composition evolved primarily towards a rapidly proliferating phenotype but with the occasional temporary appearance of slower-growing cell clones; in terms of tumor size, these fluctuations corresponded to temporary decreases in the total population size. A possible clinical manifestation of this effect could be saltatory tumor growth, which can be observed in several tumor types, such as hemangioblastomas [18]–[20]. In the proposed model, the concentration of metabolically available carbon was taken to be constant, which can be interpreted as similar to micro-environmental conditions in the brain. The model thus proposes an explanatory mechanism for saltatory tumor growth as being driven by cell heterogeneity within the tumor. Although this effect originated due to increased nutrient availability, it is possible that these fluctuations that resulted from changes in cell composition could be caused by other micro-environmental changes that are not yet understood.

The effects of up-regulation of nutrient transporters on the direction in which the cell population evolved were also investigated in order to evaluate whether therapeutically targeting nutrient transporters could be expected to significantly affect tumor growth dynamics. Results obtained from numerical calculations suggested that increasing nutrient uptake rates of the s-clones did not give them a significant competitive advantage, since their growth was also limited by carbon availability, which in this model was held constant. However, unexpectedly, increasing phosphorus uptake rates of r-clones did not confer them a competitive advantage (Figure 5). One possible explanation for this counter-intuitive result could be activation of a form of ‘futile metabolism’, where a cell is forced to spend available energy sources to rid itself of excess phosphorus instead of proliferating, if the appropriate C∶P ratio necessary for proliferation is not available in the cell micro-environment. There results suggest that targeting phosphorus transporters therapeutically could in fact be counter-productive. Instead, one should attempt to devise strategies that would ‘encourage competition’ between tumor cells, promoting utilization of resources that are available to tumor cells for intra-tumor competition, as opposed to rapid proliferation or development of therapeutic resistance [21].

Next, the effects of variation in growth rates for different cell types were evaluated. Through numerical calculations, it was observed that while population composition with respect to each strategy can vary, depending on which clone type has a higher growth rate, the final population size could be the same regardless of population composition (Figure 5). The significance of this observation lies in the fact that the entire cell population may respond very differently to changes in the micro-environment depending not on its size but on its initial composition.

Next, the effects of changes in the micro-environment on population composition when different clones are present in different proportions at the initial time moment were evaluated. It could be observed that under the same set of micro-environmental conditions, the evolutionary dynamics of the tumor as a whole is dependent on the proportion of r-clones vs. s-clones initially present in the population (Figure 7). More specifically, the more skewed the initial distribution of clones was to 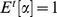 (being dominated by s-clones), the more extracellular phosphorus was required to shift population composition towards a more rapidly proliferating phenotype. This hysteretic effect is due to phenotypic heterogeneity within the tumor: if the cell population were homogeneous, a micro-environmental perturbation would have had a clear-cut bifurcation point, and predicting exactly when the population would start evolving towards a particular strategy would be possible. However, since the population is heterogeneous, population composition is a part of the environment; consequently, the fitness of each cell is affected not only by resource availability or by the cell's intrinsic properties, but also by the quantity and intrinsic properties of other cells. Consequently, the population as a whole may respond differently to the same micro-environmental perturbation, implying that any predictions about system dynamics that are made without taking into account population heterogeneity are likely to be incorrect. Therefore, in order to be able to influence the evolution of the system through micro-environmental manipulations, not only the properties of the individual cells need to be understood, but also the composition of the cell population as a whole.

The observed hysteretic effect could also account for variable effectiveness of similar treatments for similar cancer types in different patients, since treatment efficacy would be determined not only by tumor type and the current state of the micro-environment but also by tumor composition, which should be evaluated with respect to an appropriate metric (within the context of the proposed model, it is resource allocation; different metrics could be applicable for different tumors).

### Tumor dormancy

One possible manifestation of what has been termed here ‘s-clones’ is tumor dormancy. Crocker et al. [22] make the following distinction between the two types of cancer cells that may be classified as dormant: solitary dormant cells that are believed to be quiescent, defined by lack of both proliferation and apoptosis; and micro-metastatic dormant cells characterized not by the absence of proliferation and apoptosis but by their balance. It is the quiescent cells that could be interpreted within the frameworks of the proposed model as s-clones.

Tumor cells can stay dormant throughout a person's lifetime, and the triggers that would cause a switch to activity are not yet fully understood. Several mechanisms have been proposed to explain this transition from indolence to active proliferation [22]–[24]. It has been proposed that the cells can be kept in the dormant state by the immune system [25], [26] or by the lack of angiogenesis [24], [27], [28], and could switch to a rapidly proliferating state due to surgery [29] or trauma [28] that would disturb the micro-environment. The proposed model suggests that tumor cells could also progress out of the dormant state as a result of nutrition and specifically, due to the relative amounts of carbon and phosphorus in a person's diet.

### Conclusions

Tumors are evolving systems that can often adapt to any changes in their micro-environment. Acquired therapeutic resistance and disease recurrence are natural consequences of tumor heterogeneity as cytotoxic therapies wipe out the numerous more-susceptible cancer cells, leaving small subpopulations of resistant cells to grow and expand. However, one can try to ‘harness’ tumor heterogeneity for therapeutic purposes instead of attempting to eliminate it. It is conceivable that creating an environment which favors slower-growing clones might create a delay in disease progression, ultimately resulting in prolonged patient survival and better quality of life. For instance, some murine models suggest that maintaining a tumor at a constant size using minimal necessary (as opposed to maximal tolerable) chemotherapy substantially increased survival time of tumorous mice [21]. So perhaps a combination of moderate therapy, reversing adaptations that tumors created for themselves [4], such as neutralizing glycolysis-induced acidic micro-environment [30] to prevent metastatic progression, and controlling phosphorus and carbon availability in the tumor micro-environment might in fact promote survival and increase quality of life for cancer patients better than can be achieved with intensive cytotoxic therapies.

## Supporting Information

Appendix S1 (PDF)
