# Supplementary material for: Biological Stoichiometry in Tumor Micro-environments
Source: PLoS One. 2013 Jan 22;8(1):e51844. doi: 10.1371/journal.pone.0051844 (PMC3551943; doi:10.1371/journal.pone.0051844)
Supplement: Appendix S1 — (PDF) [file pone.0051844.s001.pdf]

Appendix S1

S1.1 The equation for the rate of change of the concentration of extracellular phosphorus.

Let $P_{ex}(t)$ be the concentration of phosphorus in the tumor interstitium and therefore available for cells to absorb. We assume that interstitial volume is a constant proportion of total tumor volume and scale our measure of concentration such that $P_{ex}^{tot}(t) = P_{ex}(t)N(t)$ is the absolute amount of interstitial P (recall that $N(t) = \int_0^1 x_\alpha(t)d\alpha$ is the total volume occupied by all the tumor cell clones $x_\alpha(t)$, where α accounts for the proportion of slowly proliferating (s-clones) in the total tumor).

Arterial P delivery occurs at rate $g_2(P_0 - P_{ex}(t))$, where P_0 is the P concentration in arterial blood, and g_2 is the capillary permeability constant. The term is chosen based on standard chemostat models. Interstitial phosphorus is absorbed by cells and is converted to intracellular phosphorus at rate $m(P_{ex}(t) - P_{in}(t))\frac{N(t)}{k_2+P_{ex}(t)-P_{in}(t)}$, where $P_{in}(t)$ is the intracellular P concentraiton. The term $m := m_p(1-E^t[\alpha]) + m_s E^t[\alpha]$, where $E^t[\alpha]$ is the mean value of the partition strategy α in the cell population (see main text for derivation), measures the per capita uptake rate of P throughout the tumor. Phosphorus is also released from cells that die. Therefore, the rate of change of the absolute amount of interstitial P is described by the following equation:

$$\frac{\mathrm{d}P_{ex}^{tot}(t)}{\mathrm{d}t} = \frac{\mathrm{d}P_{ex}(t)}{\mathrm{d}t}N(t) + P_{ex}(t)\frac{\mathrm{d}N(t)}{\mathrm{d}t} \tag{1}$$

$$= g_2 N(t)(P_0 - P_{ex}(t)) - mN(t)\frac{P_{ex}(t) - P_{in}(t)}{k_2 + P_{ex}(t) - P_{in}(t)} + dN(t)P_{in}(t) \tag{2}$$

and the dynamics of P_{ex} is then governed by:

$$\frac{dP_{ex}}{dt} = g_2(P_0 - P_{ex}(t)) - m\frac{P_{ex}(t) - P_{in}(t)}{k_2 + P_{ex}(t) - P_{in}(t)} + dP_{in}(t) - P_{ex}(t)E^t[F], \tag{3}$$

where

$$E^t[F] := \frac{N'(t)}{N(t)} = E^t[\alpha]r_s\left(1 - \frac{N(t)}{z(t)}\right) + (1 - E^t[\alpha])r_p\left(\frac{z(t)}{N(t) + \xi z(t)^2} - d\right), \tag{4}$$

and $z(t) = h\frac{C_{in}P_{in}(t)}{C_{in}+P_{in}(t)}$ (see main text for the derivation of both $E^t[F]$ and$z(t)$) .

S1.2 The equation for rate of change of concentration of intracellular phosphorus.

The total amount of intracellular phosphorus among all cells of type α is $P_{in}^{tot}(t) = P_{in}(t)N(t)$, where $P_{in}(t)$ is the concentration of phosphorus per cell volume. As follows from the previous equation, interstitial phosphorus is absorbed by cells and is converted to intracellular phosphorus at a rate $m(P_{ex}(t) - P_{in}(t))\frac{N(t)}{k_2+P_{ex}(t)-P_{in}(t)}$. Noticeably, intracellular phosphorus $P_{in}(t)$ is not consumed irreversibly as fuel but is used for biosynthesis, particularly of ribosomes. Therefore, the rate of change of the absolute amount of intracellular P is described by the following equation:

$$\frac{dP_{in}^{tot}}{dt} = mN(t)\frac{P_{ex}(t) - P_{in}(t)}{k_2 + (P_{ex}(t) - P_{in}(t))} \tag{5}$$

Since

$$\frac{dP_{in}^{tot}(t)}{dt} = \frac{dP_{in}(t)N(t)}{dt} = \frac{dP_{in}(t)}{dt}N(t) + P_{in}(t)\frac{dN(t)}{dt}, \tag{6}$$

the dynamics of $P_{in}(t)$ is given by

$$\frac{dP_{in}}{dt} = m\frac{P_{ex}(t) - P_{in}(t)}{k_2 + (P_{ex}(t) - P_{in}(t))} - P_{in}(t)E^t[F], \tag{7}$$

where $E^t[F]$ is defined above.
